# JMJD5: a multifunctional regulator in development, homeostasis, and cancer

**DOI:** 10.3389/fcell.2026.1818070

**Published:** 2026-05-19

**Authors:** Jing He, Haohao Wei, Yaqiong Hu, Guiling Liu

**Affiliations:** School of Basic Medicine, Gannan Medical University, Ganzhou, Jiangxi, China

**Keywords:** demethylase, JMJD5, oncogene, prognosis, tumor suppressor

## Abstract

JmjC-domain-containing oxygenases are conserved epigenetic regulators whose dysfunction is closely associated with various human diseases. JMJD5, a key member of the JmjC-Domain-Only subfamily, has multiple enzymatic activities and modulates various vital physiological and pathological processes via catalytic and non-catalytic pathways. JMJD5 also displays striking tissue-specific duality in cancer: it is frequently overexpressed and acts as an oncogene in breast, prostate, and oral squamous cell carcinomas, yet is downregulated and functions as a tumor suppressor in lung, hepatocellular, and pancreatic cancers. This functional heterogeneity is closely linked to its interacting protein networks and the tumor microenvironment. Pan-cancer analyses further reveal that JMJD5 expression correlates with patient prognosis and disease progression in multiple malignancies, highlighting its potential as a prognostic biomarker. This review systematically integrates current knowledge on JMJD5 structure, enzymatic activities, physiological functions, and cancer-associated mechanisms, identifies critical research gaps, and discusses future directions for translating JMJD5 into clinical applications.

## 1 Introduction

Cancer remains a formidable global public health challenge, with persistently rising incidence and mortality rates that threaten human health and burden socioeconomic development (Han et al., 2024; Dee et al., 2025; Siegel et al., 2026). Tumorigenesis is not driven by isolated genetic mutations but arises from the coordinated dysregulation of cellular signaling networks and epigenetic control (Li et al., 2023). Epigenetic mechanisms (such as histone methylation, acetylation, etc.) regulate gene expression reversibly without altering DNA sequences and play pivotal roles in cancer initiation, invasion, metastasis, and drug resistance (Dawson et al., 2012; Hogg et al., 2020).

JmjC-domain-containing oxygenase superfamily, the largest group of Fe(II)/α-ketoglutarate (α-KG) dependent epigenetic modifiers, exerts diverse catalytic activities including protein demethylation, hydroxylation and proteolytic cleavage on both histone and non-histone substrates (Tsukada et al., 2006; Bonnici et al., 2026; Liu et al., 2022). These enzymes act as central regulators in fundamental biological processes such as embryonic development, tissue homeostasis maintenance, circadian rhythm regulation and cellular metabolism, while their aberrant expression and dysfunction are tightly implicated in the pathogenesis of various human diseases, especially multiple malignancies (Manni et al., 2022; Yang et al., 2022). Notably, the “JmjC-Domain-Only” subfamily (small JMJD protein family), distinguished by the absence of auxiliary regulatory domains (e.g., PHD, zinc fingers) present in canonical JmjC proteins, exhibits exceptional functional diversity despite its structural simplicity (Oh et al., 2019). As the best-characterized member of this unique subfamily, JMJD5 serves as a classic model for exploring the biological functions of small JmjC proteins. JMJD5 possesses three catalytic activities (lysine demethylation, arginine hydroxylation and methyl-dependent proteolysis) and extensive non-catalytic regulatory effects, targeting histones, transcription factors and metabolic enzymes (Oh et al., 2019; Hsia et al., 2010; Liu et al., 2017; Shen et al., 2017; Wilkins et al., 2018). Physiologically, it orchestrates cell cycle progression, mitotic fidelity, microtubule dynamics, embryonic development, circadian rhythms and bone homeostasis (Jones et al., 2010; Oh and Janknecht, 2012; Youn et al., 2012; Wu J. et al., 2016). Intriguingly, JMJD5 exerts a tissue-specific dual role in cancer: it functions as an oncogene in breast, prostate, GBM and oral squamous cell carcinomas, whereas acts as a tumor suppressor in lung, hepatocellular and pancreatic cancers (Wang et al., 2019; Qu et al., 2022; Song et al., 2022; Wang et al., 2022; Shen et al., 2023). This rare functional heterogeneity among epigenetic regulators underscores the intricate biological complexity of JMJD5.

Despite increasing research on JMJD5 in recent years, several key scientific questions remain unresolved: the heterogeneous molecular mechanisms underlying its oncogenic or tumor-suppressive roles in different cancers, the interplay between its enzymatic and non-enzymatic functions, the integration of its regulatory networks (e.g., PKM2, EGFR, p53, HIF-1α pathways), and its clinical translatability as a prognostic marker or therapeutic target. Moreover, potential crosstalk with related family members (e.g., JMJD6, JMJD7) and modulation by the tumor microenvironment warrant deeper investigation.

This review systematically summarizes the structural features, enzymatic activities, and physiological functions of JMJD5, with a focus on its dual roles in different cancers. We also evaluate its potential as a biomarker and therapeutic target, identify current research gaps, and outline future directions. By integrating mechanistic insights and clinical evidence, this work aims to provide a cohesive framework for understanding JMJD5 in tumor biology and to facilitate its translation into diagnostic and therapeutic strategies.

## 2 Structure and enzymatic activities of JMJD5

### 2.1 Structural features of JMJD5

As a significant member of the JmjC domain-containing oxygenase family, JMJD5 shares its defining structural hallmark: a conserved JmjC domain that binds Fe(II) and α-KG to form a dual-cofactor catalytic center, conferring oxygenase activity (Del Rizzo et al., 2012). This domain underlies the lysine demethylase (KDM) function common to many family members, positioning them as key epigenetic regulators (Tsukada et al., 2006; Klose et al., 2006). JMJD5 belongs to the evolutionarily distinct “JmjC-Domain-Only” subfamily (small JMJD protein family), it lacks auxiliary domains such as PHD or Zinc Fingers, which may afford greater flexibility in substrate selection and functional modulation (Oh et al., 2019).

**FIGURE 1:**
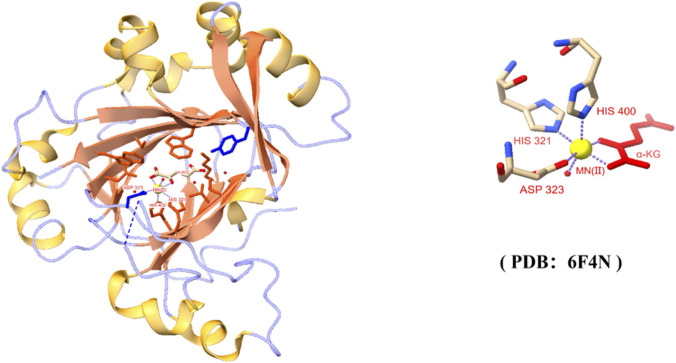
Core structure of the active site within the JmjC domain of JMJD5. (Left panel) Overall three-dimensional structure of the JMJD5 protein (ribbon diagram). Orange represents β-sheets, and yellow represents α-helices. The catalytic active center region is indicated by the right icon. Containing the key α-ketoglutarate (α-KG) binding site and the metal cofactor (Right panel) Enlarged view of the active center, showing the coordination environment of the Mn(II) ion (yellow sphere). The Mn(II) is coordinated by three highly conserved amino acid residues (His321, Asp323, His400) and the co-substrate α-KG (red) via side chains and carbonyl oxygen atoms, forming a characteristic dioxygenase catalytic center. This structure reveals the molecular basis for JMJD5 catalysis, which is dependent on Mn(II) and α-KG. Structural data were obtained from the Protein Data Bank (PDB; accession code: 6F4N) and visualized using UCSF ChimeraX.

The human *JMJD5* gene, located on chromosome 16p12.1, encodes a protein composed of 416 amino acids with a molecular weight of approximately 47 kDa. According to the NCBI and Ensembl databases, three main transcriptional variants of human JMJD5 have been annotated to date. Only one canonical protein isoform (isoform 1, 416 amino acids, ∼47 kDa) is ubiquitously expressed and functionally characterized in human cells. The other two isoforms are either tissue-specifically expressed at extremely low levels or lack the intact C-terminal JmjC catalytic domain, and their biological functions remain largely unknown. The catalytic JmjC domain resides at the C-terminus, while the N-terminus contains a nuclear localization signal (NLS) that directs JMJD5 to the nucleus (Huang et al., 2013). A functional nuclear export signal (NES) is also present in the N-terminal region (residues 10–20), which mediates the nucleocytoplasmic shuttling of JMJD5 in a cell cycle-dependent manner. During interphase, JMJD5 is predominantly localized in the nucleus, while it translocates to the cytoplasm and mitotic spindle during mitosis (Huang et al., 2013). Structurally (Figure 1), the catalytic core consists of the JmjC domain (residues 271–416) and an N-terminal extension (residues 183–270). The JmjC domain adopts a characteristic double-stranded β-helix (DSBH) fold, forming a β-barrel constructed from two antiparallel β-sheets (β7–β12–β9–β10 and β5–β6–β13–β8–β11) and surrounded by α-helices and 3_10_-helices. The N-terminal extension wraps around the β-barrel in a mixed helix-sheet topology, stabilizing the overall structure. Within the β-barrel lies the active site, which harbors a conserved pocket for Fe (II) (or Co (II)) and α-KG (2-OG). The metal ion displays an octahedral coordination geometry, ligated by three amino-acid side chains (His321, Asp323, His400), a water molecule, and the C5-carboxylate oxygen of 2-OG. 2-OG itself is anchored through a hydrogen-bond network (e.g., with Tyr272) and hydrophobic interactions (e.g., with Trp310) (Wilkins et al., 2018; Del Rizzo et al., 2012; Wang et al., 2013; Liu et al., 2018). Mutations in these key residues disrupt the catalytic center, abolishing enzymatic activity and impairing JMJD5-mediated biological functions.

### 2.2 Enzymatic activities of JMJD5

JMJD5 harbors multifunctional enzymatic activities that are well adapted to its structural simplicity, and has been confirmed to possess lysine demethylase (Hsia et al., 2010; Ishimura et al., 2012; Wang L. et al., 2025), arginine C-3 hydroxylase (Wilkins et al., 2018; Youn et al., 2012), and methyl-dependent protein hydrolase (Liu et al., 2017; Shen et al., 2017). Additionally, JMJD5 exhibits extensive regulatory functions independent of its catalytic activity. The substrate specificity, catalytic mechanisms and physiological relevance of each enzymatic activity are systematically summarized in Table 1, and detailed below.

**TABLE 1:** Enzymatic activities, substrates and target sites of JMJD5.

Enzymatic activity	Substrate type	Confirmed substrates	Target residues	Catalytic mechanism	Key physiological/Pathological functions
Lysine demethylase	Histone	H3	K36me2	Fe(II) and α-KG dependent oxidative demethylation	Cell cycle regulation (Hsia et al., 2010), circadian rhythm (Jones et al., 2010; Jones and Harmer, 2011), embryonic development (Oh and Janknecht, 2012; Ishimura et al., 2012)
	Non-histone	BECN1	K117	Same as above	Autophagy regulation in HCC (Wang L et al., 2025)
Arginine C-3 Hydroxylase	Non-histone	NFATc1	R391	Fe(II) and α-KG dependent arginine hydroxylation	Osteoclast differentiation, bone homeostasis (Youn et al., 2012)
	Non-histone	RCCD1	N-terminal arginines	Same as above	Chromosome segregation, mitosis (Wilkins et al., 2018)
Methyl-dependent protein hydrolase	Histone	H3	K9me1, R2me1	Fe(II) and α-KG dependent peptide bond cleavage	Transcriptional elongation DNA damage response (Liu et al., 2017; Shen et al., 2017)

Early studies identified JMJD5 as a specific histone H3K36me2 demethylase that removes dimethyl groups from lysine 36 via an Fe(II) and α-KG dependent oxidative mechanism, producing formaldehyde and succinate. Its reported substrates include: 1) histone H3K36me2, which regulates cell cycle genes (e.g., CCNA1, CDKN1A) and circadian clock genes (e.g., TOC1 in plants) (Hsia et al., 2010; Jones et al., 2010; Jones and Harmer, 2011); 2) non-histone BECN1 (K117), whose demethylation modulates autophagy in hepatocellular carcinoma (Wang L. et al., 2025). However, subsequent structural analyses revealed that the substrate-binding pocket of JMJD5 is approximately 2 Å narrower than that of canonical lysine demethylases (e.g., KDM4A), making it sterically impossible to accommodate a dimethylated lysine side chain (Del Rizzo et al., 2012; Wang et al., 2013). Biochemical studies using purified JMJD5 also failed to detect significant H3K36me2 demethylase activity under physiological conditions (Wilkins et al., 2018). This discrepancy remains a central debate. The early observations may arise from indirect effects (e.g., JMJD5 regulating other demethylases) or non-specific activity in overexpression systems. A detailed re-evaluation of JMJD5 enzymatic activities is provided in Section 5.3. Subsequent biochemical and structural studies confirmed that JMJD5 primarily functions as an arginine C-3 hydroxylase (Wilkins et al., 2018). It hydroxylates the guanidino group at the C-3 position, generating a hydroxyl group recognized by E3 ubiquitin ligases to target substrates for proteasomal degradation. Confirmed substrates include transcription factor NFATc1 (R391), regulating osteoclast differentiation, and chromosome condensation protein RCCD1 (N-terminal arginines), modulating mitotic chromosome segregation (Wilkins et al., 2018; Youn et al., 2012). The catalytic mechanism involves the activation of molecular oxygen by the Fe(II)/α-KG complex, which generates a highly reactive ferryl intermediate that abstracts a hydrogen atom from the C-3 position of arginine, followed by hydroxyl group transfer.

Notably, our group and others have uncovered a novel methyl-dependent protein hydrolase activity of JMJD5. It can specifically recognize methylated lysine (e.g., H3K9me) or arginine (e.g., H3R2me) residues within histones, cleaving and hydrolyzing the histone C-terminus at these modified sites. This process generates a “tailless nucleosome” structure that modulates gene expression (Liu et al., 2017; Shen et al., 2017). Indirect evidence supports this model: JMJD5 deficiency leads to abnormal nucleosome accumulation at the +1 transcription start site, resulting in transcriptional silencing of numerous genes. Moreover, JMJD5 cooperates with CDK9 to regulate the pause-release of RNA polymerase II (Pol II), suggesting that tailless nucleosomes formation facilitates transcriptional elongation (Liu et al., 2020). Importantly, this hydrolase activity is conserved in JMJD5 homologs such as JMJD6 and JMJD7 (Liu et al., 2022; Liu et al., 2017; Liu et al., 2018), indicating functional conservation within this family.

Beyond its enzyme-dependent mechanisms, JMJD5 also modulates key intracellular and extracellular proteins through non-catalytic interactions, further broadening its biological repertoire. For instance, JMJD5 negatively regulates the tumor suppressor p53 and its target genes (Huang et al., 2015); promotes nuclear translocation and activation of the glycolytic enzyme PKM2, thereby contributing to tumor metabolic reprogramming (Wang et al., 2019; Wang et al., 2014); and mediates proteasomal degradation of the epidermal growth factor receptor (EGFR), suppressing downstream EGFR signaling (Shen et al., 2023).

## 3 Biological functions of JMJD5

**FIGURE 2:**
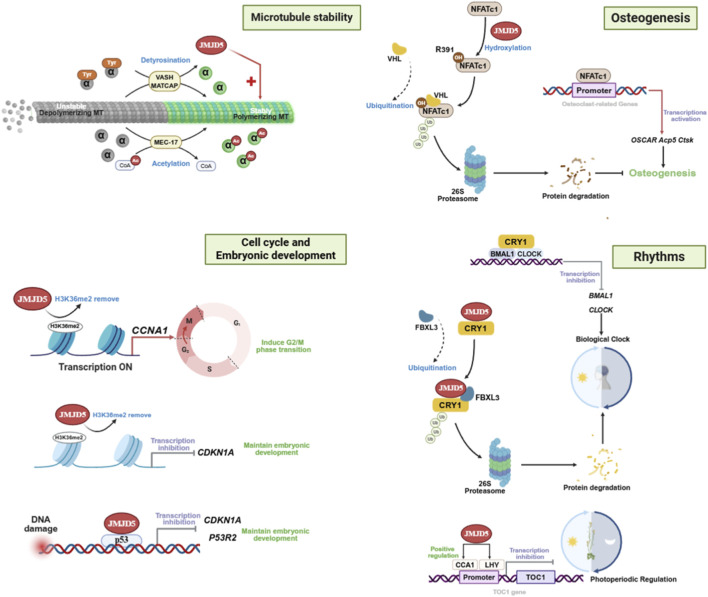
Pleiotropic regulatory functions of JMJD5 in physiological processes. (Upper Left): During microtubule stability maintenance, JMJD5 antagonizes VASH/SMTCAP-mediated detyrosination and supports MEC-17-dependent microtubule acetylation, promoting the conversion of microtubules from unstable, depolymerized states to stable, polymerized states (Lower Left): During embryonic development, JMJD5 epigenetically regulates the cell cycle by demethylating H3K36me2, activating CCNA1 transcription to induce G2/M phase transition while repressing CDKN1A to support embryonic development. In the DNA damage response, JMJD5 is recruited by p53 to activate CDKN1A and P53R2, thereby ensuring developmental integrity (Upper Right): During osteoclast differentiation, JMJD5 hydroxylates NFATc1 at residue R391, targeting it for VHL-mediated ubiquitination and subsequent proteasomal degradation. This inhibits the transcriptional activation of osteoclast-related genes (including OSCAR, Acp5, and Ctsk), thereby modulating osteoclast differentiation (Lower Right): In mammalian circadian rhythm regulation, JMJD5 interacts with CRY1 and promotes its FBXL3-mediated ubiquitination and proteasomal degradation, thereby relieving inhibition of the BMAL1/CLOCK complex and sustaining circadian rhythmicity. In plants, JMJD5 positively regulates CCA1 and LHY expression. These factors then bind to the Evening Element (EE) in the TOC1 promoter and repress its transcriptional activation, maintaining low TOC1 levels in the morning and preserving circadian stability.

As a multifunctional epigenetic regulator, JMJD5 exerts diverse biological effects through both enzymatic activity-dependent and -independent mechanisms. It governs fundamental processes including microtubule Stability regulation, cell cycle progression and embryonic development, while also playing specialized roles in circadian rhythm maintenance and skeletal homeostasis (Figure 2). Understanding the mechanistic basis of JMJD5 function is essential for deciphering its tissue-specific roles in pathological conditions, including cancer.

### 3.1 Microtubule stability and cytoskeletal regulation

JMJD5 has been reported to directly modulate cytoskeletal dynamics by regulating microtubule stability, a function that is critical for mitosis, cell migration and intracellular transport. During interphase, JMJD5 stabilizes the microtubule-associated protein MAP1B (Wu J. et al., 2016). Thereby maintaining the acetylation and detyrosination status of cytoskeletal microtubules and enhancing microtubule stability. Specifically, JMJD5 antagonizes VASH/SMTCAP-mediated microtubule detyrosination and supports MEC-17-dependent microtubule acetylation, promoting the conversion of microtubules from unstable, depolymerized states to stable, polymerized states (Wu J. et al., 2016). During mitosis, JMJD5 translocates to the mitotic spindle and directly binds to spindle microtubules, preserving the acetylation level of α-tubulin (He et al., 2016). This ensures proper spindle assembly, correct chromosome alignment and normal interkinetochore tension. Depletion of JMJD5 leads to spindle abnormalities, chromosome missegregation and aneuploidy, which are hallmarks of cancer. Furthermore, JMJD5 deficiency sensitizes tumor cells to microtubule-destabilizing chemotherapeutic agents (e.g., paclitaxel, vincristine) by altering microtubule dynamics (Wu J. et al., 2016; He et al., 2016), suggesting that JMJD5 expression levels may predict response to these drugs in cancer patients.

### 3.2 Regulation of cell cycle

Precise cell cycle control is a prerequisite for normal cell proliferation and differentiation (Matthews et al., 2022). JMJD5 contributes to this regulation at multiple levels. Enzymatically, it functions as a histone H3K36me2 demethylase to modulate the expression of core cell cycle genes. For instance, JMJD5 activates *CCNA1* transcription by removing H3K36me2 within its coding region (Hsia et al., 2010), while repressing the cell cycle inhibitor *CDKN1A* by targeting H3K36me2 in its transcriptional region (Ishimura et al., 2012). These opposing actions collectively finetune cell cycle progression under different pathophysiological conditions.

Beyond its enzymatic role, JMJD5 plays an important role in maintaining cell proliferation by regulating p53-dependent checkpoints. As described in Section 4.2.1, the inhibitory effect of JMJD5 on p53 can negatively regulate the expression of *CDKN1A* (p21) and *p53R2*, thereby participating in cell cycle regulation under stress conditions (Huang et al., 2015).

### 3.3 Embryonic development

Consistent with its role in cell cycle regulation, JMJD5 is also indispensable for embryonic development. Jmjd5-knockout mice exhibit severe growth retardation and embryonic or early postnatal lethality. The underlying mechanism involves upregulation of *Cdkn1a* (encoding p21), which potently inhibits embryonic cell proliferation (Oh and Janknecht, 2012; Ishimura et al., 2012; Zhu et al., 2014). However, concomitant *Cdkn1a* knockdown only partially rescues the developmental defects caused by Jmjd5 ablation (Ishimura et al., 2012), indicating that JMJD5 regulates embryogenesis through a broader network. Instead, this network likely includes interactions with cyclin complexes, regulation of additional cell cycle genes, and other unidentified mechanisms. Loss of JMJD5 disrupts the balance between proliferation and differentiation in embryonic cells, ultimately leading to developmental abnormalities.

### 3.4 Osteoclastogenesis and maintenance of bone homeostasis

A unifying function of JMJD5 across diverse physiological and pathological processes is the regulation of key protein stability through post-translational modifications. This regulatory mechanism is exemplified in osteoclastogenesis, and is also conserved in circadian rhythms regulation and cancer.

During osteoclastogenesis, differentiation and maturation are strictly dependent on the activation of the master transcription factor NFATc1, which binds to promoters of osteoclast-related genes (e.g., OSCAR, Acp5, Ctsk) to drive the osteoclastic phenotype (Kang et al., 2020; Omata et al., 2023). JMJD5 acts as a negative regulator of NFATc1 through its arginine hydroxylase activity (Youn et al., 2012). Specifically, JMJD5 catalyzes hydroxylation of arginine 391 (R391) on NFATc1, a modification that enables recognition by the E3 ubiquitin ligase VHL. This recognition targets NFATc1 for ubiquitin-proteasomal degradation, reducing its protein levels and ultimately suppressing osteoclast formation. This regulatory mechanism aligns with that by which JMJD5 mediates CRY1 degradation, revealing a common functional paradigm: JMJD5 executes specific physiological and pathological roles by regulating the stability of key proteins through post-translational modifications.

### 3.5 Regulation of circadian rhythms in animals and plants

JMJD5 also plays an evolutionarily conserved role in the circadian clock systems of both animals and plants, functioning as an integral component of the circadian machinery. In mammals, JMJD5 regulates clock protein stability through direct protein-protein interactions: it binds to the cryptochrome CRY1 and promotes its association with the E3 ubiquitin ligase FBXL3, leading to CRY1 proteasomal degradation. This degradation relieves CRY1-mediated repression of core CLOCK/BMAL1 complex, thereby modulating circadian period and phase (Saran et al., 2018). In plants (e.g., *Arabidopsis thaliana*), JMJD5 similarly contributes to photoperiod sensing and the transcriptional regulation of core circadian clock genes. JMJD5 interacts with the core clock protein TOC1 to promote the transcription of morning-expressed genes including CCA1 and LHY. Meanwhile, JMJD5 directly binds to the Evening Element (EE) within the TOC1 promoter and represses TOC1 expression. In turn, TOC1 inhibits LHY and CCA1 expression through CHE, thus forming a negative feedback loop (Jones et al., 2010; Jones and Harmer, 2011). JMJD5-mediated demethylation of histone H3K36me2 serves as a key mechanism underlying this transcriptional regulation.

## 4 JMJD5: from homeostasis to malignancy

As delineated above, JMJD5 exerts pleiotropic biological effects during normal physiological processes, highlighting its dual role as an epigenetic modifier and a regulator of protein stability. The transition from physiological homeostasis to tumorigenesis typically hinges on the aberrant modulation of core interacting networks within specific tumor microenvironments. Although JMJD5 exhibits striking tissue-specific duality—acting as either an oncogene or a tumor suppressor—its functional outputs in various malignancies frequently converge on several critical molecular axes. Among these, the JMJD5-PKM2 metabolic axis and the JMJD5-p53 signaling network stand out as paradigmatic hubs that coordinate essential processes, including metabolic reprogramming, cell cycle progression, and apoptotic evasion. Consequently, isolating and systematically summarizing these two hallmark regulatory pathways provides a cohesive mechanistic framework to decipher the complex and context-dependent roles of JMJD5 in human cancers.

### 4.1 Metabolic regulation via the JMJD5-PKM2 axis

**FIGURE 3:**
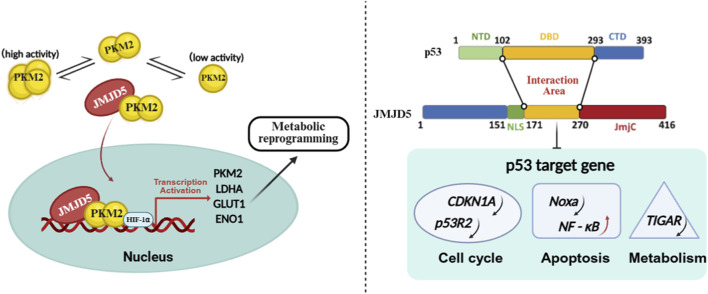
Key Molecular Axes Regulated by JMJD5. (Left) JMJD5 promotes the nuclear translocation of PKM2 dimers, where intranuclear PKM2 transcription coactivates the HIF-1α factor, driving the expression of key glycolytic enzymes (PKM2, LDHA, GLUT1, and ENO1) and inducing metabolic reprogramming (Right)The amino acid region 171–270 of JMJD5 interacts with the p53 DNA-binding domain (DBD), negatively regulating downstream target genes of p53: inhibiting cell cycle-related genes CDKN1A and p53R2; suppressing apoptosis-related factor Noxa while activating the NF-κB signaling pathway; inhibiting the key enzyme TIGAR in the pentose phosphate pathway.

Cancer cells predominantly exhibit reprogrammed energy metabolism, known as the Warburg effect, wherein they favor aerobic glycolysis over oxidative phosphorylation (Cairns et al., 2011; Li et al., 2025). As a key metabolic regulatory factor, JMJD5 modulates the Warburg effect by targeting pyruvate kinase M2 (PKM2). JMJD5 interacts physically with PKM2, regulates its oligomeric state, and facilitates nuclear translocation of PKM2. Upon entering the nucleus, PKM2 acts as a transcription coactivator for hypoxia-inducible factor-1α (HIF-1α), driving the expression of key glycolytic enzymes (including PKM2, LDHA, GLUT1, and ENO1) and ultimately inducing metabolic reprogramming to meet the energy demands required for malignant tumor progression (Figure 3). This conserved metabolic transition promotes tumor cell proliferation and invasion, establishing a consistent mechanistic foundation for the oncogenic roles of JMJD5 in breast, prostate, and glial malignancies (Wang et al., 2019; Song et al., 2022; Wang et al., 2014).

### 4.2 Signaling control via the JMJD5-p53 interaction

As a key negative regulator of the p53 signaling pathway, JMJD5 physically binds to the DNA-binding domain of p53, thereby inhibiting the recruitment of p53 to the promoter regions of its target genes. This interaction exerts broad transcriptional control over multiple biological processes(Figure 3): Inhibiting the transcriptional activation of CDKN1A (p21) and p53R2, thereby regulating cell cycle arrest in DNA damage response (Huang et al., 2015); Suppressing the activation of pro-apoptotic factors such as Noxa, blocking the initiation of programmed cell death (Oh and Janknecht, 2012); Activating of the NF-κB signaling pathway, synergistically promoting cell survival and inhibits caspase-dependent apoptosis signaling (Yao et al., 2019); Negatively regulating TIGAR (a key enzyme in the pentose phosphate pathway), reducing metabolic flux toward the pentose phosphate pathway, thereby reducing cellular damage caused by reactive oxygen species (ROS) accumulation (Liu et al., 2023). By modulating the p53 signaling network, JMJD5 functions as a molecular rheostat, playing a pivotal role in maintaining cellular homeostasis and driving malignant transformation.

## 5 JMJD5 and cancer

**FIGURE 4:**
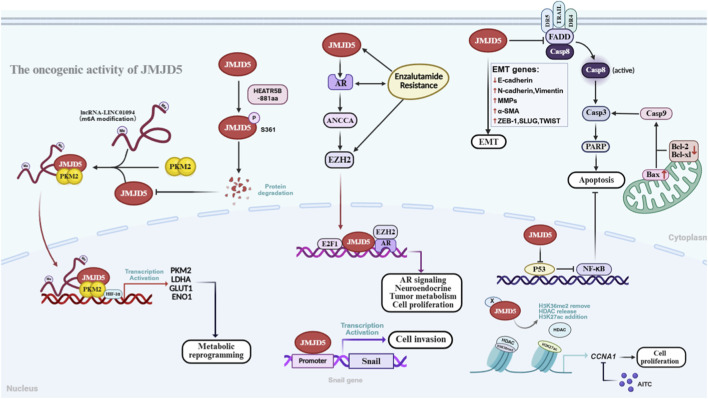
Oncogenic Functions of JMJD5 in Tumorigenesis. (Left) In breast cancer, the m6A-modified long non-coding RNA LINC01094 acts as a molecular scaffold to promote the formation of the PKM2/JMJD5 complex, further enhancing the nuclear translocation of PKM2 dimers to amplify its oncogenic effects. In GBM, the HEATR5B-881aa protein specifically phosphorylates JMJD5 at serine 361 (S361), targeting JMJD5 for proteasomal degradation and inhibiting the oncogenic activity of the JMJD5/PKM2 pathway (Upper Center) In CRPC, JMJD5 mediates the expression of neuroendocrine markers via the AR-EZH2 axis, thereby promoting the malignant progression of prostate cancer (Lower Center) In breast cancer, JMJD5 binds to the Snail promoter, activates its transcription, and thereby promotes cell migration and invasion (Upper Right) In OSCC, JMJD5 promotes cell migration and invasion by inducing the expression of EMT-related genes (E-cadherin, MMPs, α-SMA, ZEB-1). Furthermore, JMJD5 inhibits two caspase-dependent apoptotic pathways—the intrinsic pathway (internally activated by members of the B-cell leukemia/lymphoma-2 (Bcl-2) protein family) and the extrinsic pathway (externally activated by pro-apoptotic ligands interacting with specialized death receptors (DRs) on the cell surface)—via negative regulation of the p53/NF-κB axis (Lower Right) In breast cancer, JMJD5 interacts with an unknown cellular co-factor (protein X) to enhance CCNA1 transcriptional activity and promote cell proliferation. This process is achieved by removing H3K36me2 marks on CCNA1, thereby disrupting the recruitment of histone deacetylases (HDACs). Allyl isothiocyanate (AITC) can inhibit the JMJD5-CCNA1 axis, arresting the cell cycle and suppressing cell proliferation.

**FIGURE 5:**
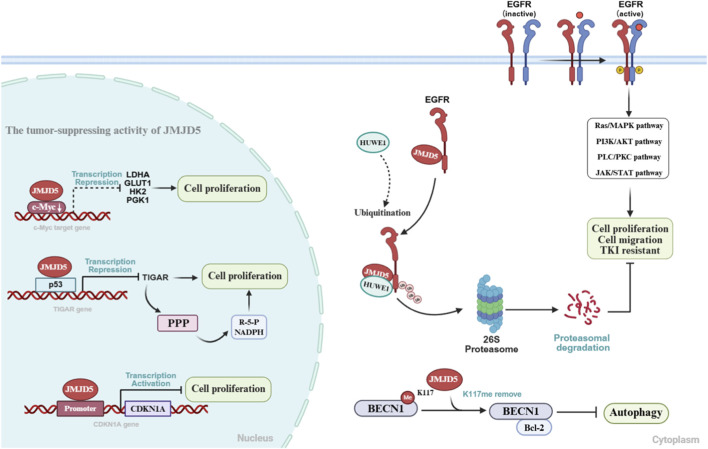
Tumor-Suppressive Functions of JMJD5 in Tumorigenesis. Nucleus (top to bottom): In pancreatic cancer, JMJD5 binds to the proto-oncogene c-Myc and downregulates its expression, thereby inhibiting the transcriptional activation of key glycolytic regulatory genes (LDHA, GLUT1, HK2, PGK1) and suppressing cell proliferation. In lung cancer, JMJD5 inhibits the metabolic flux toward the pentose phosphate pathway (PPP) and suppresses reactive oxygen species (ROS) accumulation by negatively regulating the p53-TIGAR pathway. In HCC, JMJD5 binds to the CDKN1A promoter and activates its transcription, arresting the G1/S phase of the cell cycle to inhibit cell proliferation. Cytoplasm (top to bottom): In lung cancer, JMJD5 recruits the E3 ubiquitin ligase HUWE1 to promote the proteasomal degradation of EGFR, which inhibits cell proliferation and migration while enhancing the sensitivity of lung cancer cells to tyrosine kinase inhibitors (TKIs). In HCC, JMJD5 specifically removes the methylation modification at lysine 117 (K117) of the key autophagy protein BECN1, promoting the binding of BECN1 to the anti-apoptotic protein BCL2 and inhibiting excessive autophagy activation. R-5-P:D-Ribose 5-phosphate---products of the pentose phosphate pathway.

JMJD5 plays a dual role in tumorigenesis and progression, exhibiting distinct tissue-specific functions. It is frequently overexpressed and acts as an oncogene in breast cancer, prostate cancer, oral squamous cell carcinoma (OSCC) and other malignancies (Figure 4), whereas its expression is markedly downregulated and functions as a tumor suppressor in lung cancer, hepatocellular carcinoma (HCC), and pancreatic cancer (Figure 5). This functional heterogeneity is closely linked to JMJD5 expression levels, its interacting protein networks and the tumor microenvironment. In this section, we systematically dissect the regulatory mechanisms of JMJD5 in different cancers from the dual perspectives of oncogenic and tumor-suppressive functions (Table 2).

**TABLE 2:** Roles and regulatory mechanisms of JMJD5 in various cancers.

Function	Cancer type	Regulatory molecule/Signaling pathway	Mechanism
Oncogenic	Breast Cancer	CCNA1	Removes H3Kme2 on CCNA1, enhancing transcriptional activity (Hsia et al., 2010)
		Snail	Binds *Snail* promoter, upregulating its transcription (Zhao et al., 2015)
		PKM2/HIF-1α	Promotes PKM2 nuclear translocation, enhances HIF-1α transcriptional activity, mediates metabolic reprogramming (Wang et al., 2014)
		LINC01094	Facilitates PKM2 dimerization and nuclear translocation (Wang M et al., 2025)
	OSCC	p53/NF-κB	Downregulates p53 to inhibit apoptosis; mediates NF-κB nuclear translocation to induce EMT and promote invasion (Yao et al., 2019)
		MTA1	Compound interferes with MTA1-JMJD5 axis, inhibiting cell proliferation (Yang et al., 2020)
		CCNA1	Compound inhibits JMJD5-CCNA1 axis, arresting cell cycle (Hsieh et al., 2023)
	Prostate Cancer	AR, PKM2	Acts as AR/PKM2 co-activator, upregulates ANCCA-EZH2 pathway, promoting castration resistance (Wang et al., 2019)
	Glioblastoma	HEATR5B-881aa (encoded by circHEATR5B)	HEATR5B-881aa phosphorylates JMJD5 at S361, promoting its degradation and inversely inhibiting glycolysis and proliferation (Song et al., 2022)
	Colon Cancer	Unclear	Promote cell proliferation, migration, and invasion capabilities [Zhang et al., 2015]
	Meningioma	Unclear	Poor prognosis (Lee et al., 2015)
Tumor Suppressor	Lung Cancer	EGFR	Recruits HUWE1 to promote EGFR proteasomal degradation, inhibits EGFR-related signaling pathways (non-enzymatic) (Shen et al., 2023)
		P53/TIGAR	Negatively regulates p53-TIGAR axis, mediates metabolic flux shift, inhibits ROS accumulation (non-enzymatic) (Liu et al., 2023)
	HCC	CDKN1A	Binds CDKN1A promoter to activate transcription, arresting G1/S phase (non-enzymatic) (Wu B. H. et al., 2016)
		Beclin1 (BECN1)	Demethylates BECN1 at K117, promotes BECN1-BCL2 binding, inhibits autophagy (enzymatic) (Wang L et al., 2025)
	Pancreatic Cancer	c-Myc	Downregulates c-Myc, inhibiting glycolysis and cell proliferation (Wang et al., 2022)

### 5.1 Oncogenic functions and mechanisms of JMJD5

#### 5.1.1 Oncogenic role in breast cancer

JMJD5 promotes breast cancer progression through multiple synergistic mechanisms. Epigenetically, it catalyzes H3K36me2 demethylation within the *CCNA1* coding region, enhancing its transcription, inducing G2/M phase transition, and thereby driving cancer cell proliferation (Hsia et al., 2010). Through a non-enzymatic mechanism, JMJD5 acts as a transcriptional co-activator that binds the promoter of *Snail*, a key epithelial-mesenchymal transition (EMT) transcription factor, activating its expression and enhancing cancer cell invasion and migration (Zhao et al., 2015). In terms of metabolic reprogramming, JMJD5 remodels glucose metabolism in breast cancer cells by regulating the PKM2-HIF-1α metabolic axis (see Section 4.1 for details) (Wang et al., 2014). Notably, the enzymatically inactive JMJD5 mutant (H321A) retains the ability to activate HIF-1α, indicating that this process is independent of JMJD5 catalytic activity (Wang et al., 2014; Schoepflin et al., 2017).

Furthermore, another study found that long non-coding RNA LINC01094 can serve as a “flexible scaffold” to facilitate the formation of the PKM2/JMJD5 complex and enhance PKM2 nuclear translocation (Wang M. et al., 2025). This finding further corroborates the direct interaction between JMJD5 and PKM2 as well as the functional correlation between them.

Additionally, soybean extract has been suggested to suppress breast cancer progression by downregulating JMJD5 expression, yet this conclusion awaits further mechanistic validation (Wang et al., 2018). To date, bioinformatic analyses have linked high JMJD5 expression to a favorable prognosis in breast cancer patients (Li et al., 2021); however, direct experimental evidence supporting this correlation remains insufficient. Overall, current evidence strongly indicates that JMJD5 functions as an oncogenic driver in breast cancer and may serve as a promising therapeutic target.

#### 5.1.2 Oncogenic role in oral squamous cell carcinoma

JMJD5 is highly expressed in Oral Squamous Cell Carcinoma (OSCC) tissues and cell lines, and its elevated expression serves as a prognostic indicator of poor outcomes in OSCC patients (Yao et al., 2019; Yang et al., 2020; Hsieh et al., 2023). Its oncogenic functions primarily involve inhibition of apoptosis and promotion of cell invasion. As described in Section 4.2, JMJD5 downregulates p53 to activate the NF-κB pathway, which suppresses both intrinsic and extrinsic caspase-dependent apoptotic signaling and reduces OSCC cell apoptosis (Yao et al., 2019). Concurrently, JMJD5 enhances OSCC cell migration and invasion by inducing the expression of epithelial-mesenchymal transition (EMT)-related genes, including E-cadherin, matrix metalloproteinases (MMPs), α-smooth muscle actin (α-SMA), and zinc finger E-box-binding homeobox 1 (ZEB-1) (Yao et al., 2019).

Furthermore, plant-derived natural compounds exert anti-OSCC effects by targeting JMJD5: silibinin acts by disrupting the MTA1/JMJD5 regulatory axis (Yang et al., 2020), while allyl isothiocyanate functions through inhibiting the JMJD5/CCNA1 axis (Hsieh et al., 2023), both compounds suppress OSCC cell proliferation and tumor growth *in vitro* and *in vivo*. However, whether JMJD5-mediated regulation of CCNA1 in OSCC depends on its enzymatic activity remains unclear and requires further experimental validation.

#### 5.1.3 Oncogenic role in prostate cancer

In prostate cancer, JMJD5 acts as a dual co-activator for the androgen receptor (AR) and PKM2, playing a critical role in the development of castration-resistant prostate cancer (CRPC). On one hand, JMJD5 drives aerobic glycolysis by regulating the PKM2-HIF-1α metabolic axis (see Section 4.1 for detail), driving aerobic glycolysis to support CRPC cell proliferation under low-androgen conditions (Wang et al., 2019). On the other hand, JMJD5 interacts with AR to enhance its transcriptional activity. Both proteins are co-recruited to the enhancer region of the *ANCCA* locus, upregulating *ANCCA* and activating its downstream target EZH2, a core component of the PRC2 complex that represses tumor suppressor genes, thereby promoting prostate cancer progression (Wang et al., 2019). TCGA data show a positive correlation between JMJD5 and EZH2 expression in prostate cancer. Knockdown of JMJD5 or treatment with EZH2 inhibitors reverses the neuroendocrine phenotype of CRPC cells and restores sensitivity to the AR inhibitor enzalutamide (Wang et al., 2019), suggesting JMJD5 as a potential therapeutic target for CRPC.

#### 5.1.4 Oncogenic role in glioblastoma multiforme

In glioblastoma multiforme (GBM), Song et al. reported that JMJD5 is highly expressed in both tumor tissues and cell lines, with expression levels positively correlated with pathological grade. JMJD5 promotes glycolysis and cell proliferation by enhancing PKM2 enzymatic activity (see Section 4.1 for details) (Song et al., 2022). Notably, this study identified that HEATR5B-881aa, a protein encoded by the circular RNA circHEATR5B, specifically phosphorylates JMJD5 at serine 361 (S361) (Song et al., 2022). This phosphorylation targets JMJD5 for proteasomal degradation, providing a novel regulatory mechanism and potential therapeutic target for GBM. Moreover, this work represents the first demonstration of JMJD5 phosphorylation as a post-translational regulatory mechanism, opening new avenues for investigating how such modifications modulate JMJD5 biological functions.

#### 5.1.5 Oncogenic role in other cancers

Beyond the tumor types discussed above, JMJD5 also exerts oncogenic effects in colon cancer and atypical meningioma. In colon cancer, Zhang et al. demonstrated that JMJD5 is highly expressed in tumor tissues and correlates with poor patient prognosis. Depletion of JMJD5 inhibits colon cancer cell proliferation, migration, and invasion, though the underlying regulatory mechanisms remain to be elucidated (Zhang et al., 2015). In atypical meningioma, survival analyses showed that patients with low JMJD5 expression exhibit lower recurrence rates and longer overall survival, implicating JMJD5 in the malignant progression of this tumor type. However, mechanistic investigations in this field are still scarce (Lee et al., 2015).

### 5.2 Tumor-suppressive functions of JMJD5

#### 5.2.1 Tumor-suppressive role in lung cancer

Multiple studies from our group have demonstrated that JMJD5 is downregulated in lung cancer tissues and cell lines, and its low expression correlates with poor patient prognosis. Functionally, JMJD5 inhibits lung cancer progression through diverse mechanisms that are largely independent of its enzymatic activity.

On the one hand, as described in Section 4.2, JMJD5 downregulates TIGAR expression through p53-dependent mechanisms, thereby inhibiting malignant tumor progression (Liu et al., 2023). On the other hand, JMJD5 negatively regulates the oncogenic driver EGFR, further validating the classical regulatory mechanism of executing biological functions through the precise modulation of key protein stability. Specifically, JMJD5 binds to the tyrosine kinase (TK) domain of EGFR, inhibiting its activation and downstream signaling pathways (e.g., ERK, PI3K/AKT). Moreover, JMJD5 recruits the E3 ubiquitin ligase HUWE1 to promote the proteasomal degradation of both wild-type and drug-resistant mutant EGFR, thereby enhancing the sensitivity of lung cancer cells to EGFR tyrosine kinase inhibitors (Shen et al., 2023).

Notably, our team was the first to demonstrate that JMJD5 can be secreted extracellularly via exosomes, exosomal JMJD5 inhibits lung cancer cell proliferation and migration both *in vitro* and *in vivo* (Shen et al., 2023). This finding provides a mechanistic explanation for the low JMJD5 protein levels detected in pleural effusions from lung cancer patients (Wang et al., 2012), more importantly, proposes a potential therapeutic strategy involving exosome-mediated JMJD5 delivery.

Although one study reported an association between high JMJD5 expression and poor prognosis in lung cancer patients receiving platinum-based chemotherapy (Xiang et al., 2019), contradicting the tumor-suppressive role, the underlying mechanisms were not explored. Further validation and mechanistic investigation are therefore warranted.

#### 5.2.2 Tumor-suppressive role in hepatocellular carcinoma

JMJD5 is specifically highly expressed in normal liver tissues, suggesting its physiological importance in maintaining liver function. In contrast, JMJD5 expression is significantly downregulated in Hepatocellular carcinoma (HCC) tissues, with this dysregulation primarily attributed to epigenetic mechanisms (Qu et al., 2022). Ahmed et al. identified hypermethylation at specific CpG sites (e.g., cg02871891, cg03101936) within the JMJD5 promoter in HCC, which directly suppresses its transcription (Ahmed et al., 2024). Concurrently, Wu et al. confirmed via chromatin immunoprecipitation (ChIP) that active histone modifications (e.g., H3Kac, H3K27ac, H3K4me3) are decreased, whereas repressive markers (H3Kme3, H3K27me3) are increased at the JMJD5 promoter, contributing to its transcriptional silencing (Wu B. H. et al., 2016). However, the interplay between these two epigenetic regulatory mechanisms requires systematic investigation.

Functionally, JMJD5 exerts tumor-suppressive effects through both enzymatic and non-enzymatic mechanisms. It directly binds the CDKN1A promoter and activates its transcription in a p53-independent manner, upregulating p21 expression and inhibiting HCC cell proliferation—a process independent of its demethylase activity (Wu B. H. et al., 2016). Additionally, JMJD5 functions as a specific demethylase for the autophagy protein BECN1, removing the methylation mark at lysine 117 (K117), and promotes BECN1 binding to the anti-apoptotic protein BCL2, and thus inhibiting excessive autophagy activation. Conversely, JMJD5 downregulation in HCC leads to accumulation of BECN1 K117 methylation, aberrant autophagy activation, and tumor progression (Wang L. et al., 2025).

#### 5.2.3 Tumor-suppressive role in pancreatic cancer

JMJD5 expression is downregulated in pancreatic cancer tissues, and its low expression is associated with poor patient prognosis. Mechanistically, JMJD5 negatively regulates the expression of the proto-oncogene c-Myc, inhibiting the activation of its key target glycolytic enzyme (such as LDHA), thereby reducing glucose uptake and lactate production in pancreatic cancer cells. Conversely, deficiency of JMJD5 enhances c-Myc expression, promoting glycolysis and cell proliferation (Wang et al., 2022). However, the precise mechanism by which JMJD5 regulates c-Myc and whether this process depends on its enzymatic activity remain to be determined. Notably, JMJD5 promotes glycolysis in breast cancer through the PKM2 axis (Wang et al., 2019; Wang et al., 2014), whereas it inhibits glycolysis in pancreatic cancer via the c-Myc axis. This pronounced tissue-specific functional polarization is one of the hallmark features in studies of JMJD5.

### 5.3 Expression and prognostic analysis of JMJD5 in pan-cancer

The above mechanistic studies demonstrate that JMJD5 exerts diametrically opposed functions in different cancer types through distinct enzymatic and non-enzymatic mechanisms. To systematically validate these findings across a broader range of malignancies and evaluate the clinical relevance of JMJD5, we performed a comprehensive pan-cancer analysis using transcriptomic data from The Cancer Genome Atlas (TCGA) database. This analysis not only confirms the tissue-specific expression patterns of JMJD5 observed in individual cancer studies, but also reveals novel correlations between JMJD5 expression and patient prognosis, disease progression, and clinicopathological features across 33 cancer types.

**FIGURE 6:**
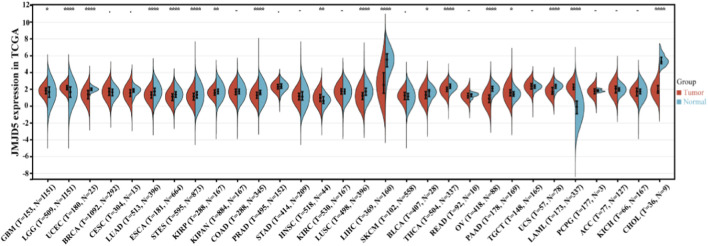
Pan-cancer analysis of JMJD5 expression. JMJD5 transcript levels in various cancer types from the TCGA cohorts were analyzed using the SangerBox platform (http://sangerbox.com/home.html). *p < 0.05, **p < 0.01, ***p < 0.001, ****p < 0.0001.

To systematically characterize the expression patterns and clinical relevance of JMJD5 across diverse tumor types, we analyzed JMJD5 transcript levels in multiple cancer cohorts from the TCGA database using the SangerBox platform (Figure 6). Our results revealed striking tumor-type specificity in JMJD5 expression. Compared with matched normal tissues, JMJD5 was upregulated in glioblastoma multiforme (GBM), lower-grade glioma (LGG), head and neck squamous cell carcinoma (HNSC), pancreatic adenocarcinoma (PAAD), and acute myeloid leukemia (LAML), with the most pronounced elevation observed in LAML. Conversely, JMJD5 was downregulated in uterine corpus endometrial carcinoma (UCEC), lung adenocarcinoma (LUAD), esophageal carcinoma (ESCA), stomach and esophageal carcinoma (STES), kidney renal papillary cell carcinoma (KIRP), colon adenocarcinoma (COAD), lung squamous cell carcinoma (LUSC), liver hepatocellular carcinoma (LIHC), bladder urothelial carcinoma (BLCA), thyroid carcinoma (THCA), ovarian cancer (OV), uterine carcinosarcoma (UCS), and cholangiocarcinoma (CHOL), with the most marked reductions detected in LIHC and CHOL.

**FIGURE 7:**
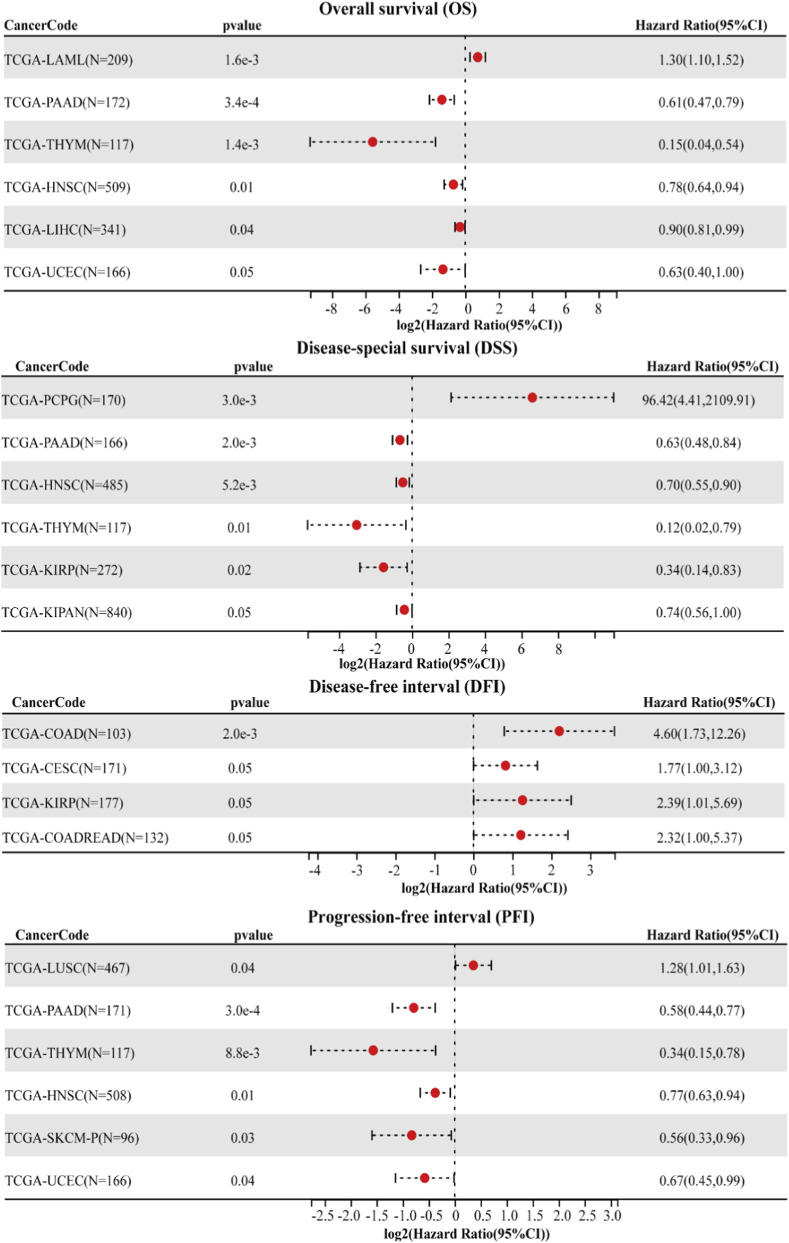
Pan-cancer analysis of survival associations for JMJD5 expression. Correlation between JMJD5 expression levels and patient overall survival (OS), disease-specific survival (DSS), disease-free interval (DFI), and progression-free interval (PFI) across multiple cancer types from TCGA cohorts was analyzed using the SangerBox platform. Red dots represent the estimated hazard ratio (HR), with horizontal lines indicating the 95% confidence interval (CI). Tumor types are listed on the left, with corresponding P-value and HR (95% CI) shown for each survival endpoint. A pvalue<0.05 was considered statistically significant.

Survival analyses further uncovered complex associations between JMJD5 expression and patient outcomes (Figure 7). For overall survival (OS), high JMJD5 expression correlated with poor prognosis in LAML patients (P < 0.05), but was associated with favorable OS in PAAD, thymoma (THYM), HNSC, LIHC, and UCEC (P < 0.05). In disease-specific survival (DSS), elevated JMJD5 levels predicted longer survival in PAAD, HNSC, THYM, KIRP, and kidney renal clear cell and papillary cell carcinoma (KIPAN) (P < 0.05), yet were linked to shorter DSS in pheochromocytoma and paraganglioma (PCPG) (P < 0.05). Regarding progression-free interval (PFI), high JMJD5 expression correlated with prolonged PFI in COAD, cervical squamous cell carcinoma and endocervical adenocarcinoma (CESC), KIRP, and colon adenocarcinoma and rectal adenocarcinoma (COADREAD), Similarly, elevated JMJD5 was associated with extended disease-free interval (DFI) in PAAD, THYM, HNSC, skin cutaneous melanoma (SKCM), and UCEC, but with shortened DFI in LUSC (P < 0.05). These findings suggest that JMJD5 may serve as a prognostic biomarker in multiple cancer types.

**FIGURE 8:**
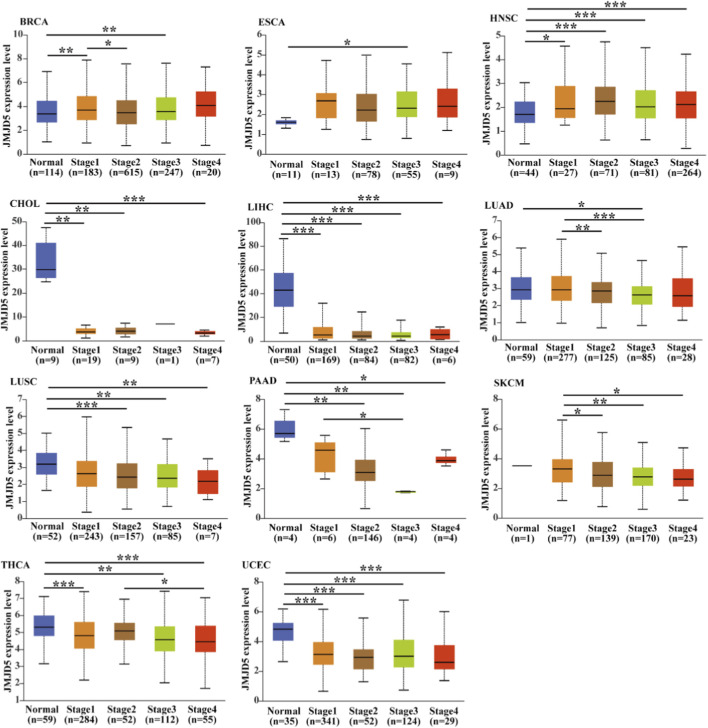
Pan-cancer analysis of JMJD5 expression across clinical stages. JMJD5 expression levels in multiple tumor types from TCGA cohorts were analyzed across different clinical stages (Normal, Stage I–IV) using the UALCAN platform (https://ualcan.path.uab.edu/). *p < 0.05, **p < 0.01, ***p < 0.001, ****p < 0.0001.

Analysis of JMJD5 expression across pathological stages revealed that JMJD5 levels increased with advancing tumor stage in breast invasive carcinoma (BRCA), ESCA, and HNSC. In contrast, JMJD5 expression declined during disease progression in CHOL, LIHC, LUAD, LUSC, PAAD, SKCM, THCA, and UCEC, with the most pronounced decreases observed in CHOL, LIHC, and PAAD (Figure 8). These data indicate that JMJD5 expression may be implicated in the disease progression of multiple malignancies.

Notably, our analysis results differ from some previously published findings regarding JMJD5 expression, prognosis, and stage correlation in certain cancer types. For instance, in LUSC, TCGA data showed that high JMJD5 expression was associated with shorter DFI, suggesting a potential adverse prognostic role, a finding that appears inconsistent with its downregulation in lung cancer tissues and its reported tumor-suppressive function (Shen et al., 2023; Liu et al., 2023). Similarly in COAD, TCGA analysis revealed that reduced JMJD5 expression in tumor tissues correlated with prolonged PFI, whereas prior studies reported elevated JMJD5 expression as a predictor of poor prognosis (Zhang et al., 2015). These discrepancies may arise from differences in sample sources, analytical platforms, cohort characteristics, and therapeutic backgrounds. They may also reflect the functional complexity of JMJD5: within the same cancer type, JMJD5 may exert both oncogenic and tumor-suppressive effects through distinct mechanisms, with the net outcome shaped by the specific tumor microenvironment, molecular subtype, and genetic background.

Collectively, JMJD5 integrates multiple core signaling pathways into a unified regulatory network that governs cell proliferation, apoptosis, metabolism, invasion, and differentiation. These pathways are not isolated but exhibit extensive crosstalk. For example, the PKM2-HIF-1α metabolic axis intersects with the p53-CDKN1A cell cycle axis: JMJD5-mediated PKM2 nuclear translocation enhances HIF-1α activity, which in turn represses p53 expression, further promoting cell cycle progression. Similarly, the EGFR-HUWE1 degradation axis crosstalks with the c-Myc glycolytic axis in lung and pancreatic cancers: JMJD5-induced EGFR degradation inhibits downstream ERK signaling, which reduces c-Myc phosphorylation and stability, thereby suppressing aerobic glycolysis. In prostate cancer, the AR-EZH2 epigenetic axis and the PKM2-HIF-1α metabolic axis converge at JMJD5, which acts as a dual co-activator for both AR and PKM2, coordinating androgen signaling and metabolic reprogramming to drive castration resistance. The functional outcome of JMJD5 in a specific tissue is ultimately determined by the relative strength of these interconnected signaling pathways and the composition of its interacting protein network.

## 6 Conclusion and future perspectives

This review systematically summarizes the structural characteristics (Figure 1), multifunctional enzymatic activities (Table 1), and complex regulatory roles of JMJD5 in physiological processes and tumorigenesis (Figures 2, 4, 5). By integrating data from public databases, we clarify the differential expression of JMJD5 across various cancer types and analyze the correlation between its expression and cancer patients’ survival, prognosis, and clinicopathological features (Figures 6–8). The core findings are as follows: As a member of the small JMJD subfamily, JMJD5 features a conserved JmjC domain as its functional core, harboring lysine demethylase (Hsia et al., 2010; Ishimura et al., 2012; Wang L. et al., 2025), arginine hydroxylase (Wilkins et al., 2018; Youn et al., 2012), and methyl-dependent protein hydrolase activities (Liu et al., 2017; Shen et al., 2017), as well as non-enzymatic regulatory modes. A detailed re-evaluation of JMJD5’s enzymatic activities and the proposed demethylation-driven hierarchical catalytic model is presented in Section 6.3. This diversity in enzymatic activities and the demethylation-driven sequential regulatory mechanisms form the core molecular basis for its involvement in multiple physiological and pathological processes. Current research confirms that JMJD5 plays a central regulatory role in mammalian embryonic development (Oh and Janknecht, 2012; Ishimura et al., 2012; Zhu et al., 2014), circadian rhythm maintenance in plants and animals (Jones et al., 2010; Saran et al., 2018), osteoclastogenesis (Youn et al., 2012), and tumor progression. Notably, JMJD5 exhibits distinct tissue-specific functions in tumors: it is highly expressed and acts as an oncogene in cancers such as breast and prostate cancer (Wang et al., 2019; Song et al., 2022; Wang M. et al., 2025; Hsieh et al., 2023), while being lowly expressed with tumor-suppressive effects in lung cancer and hepatocellular carcinoma (Qu et al., 2022; Wang et al., 2022; Shen et al., 2023; Liu et al., 2023). Its expression level is closely correlated with patients’ prognosis and clinical stage across various cancers, positioning it as a potential therapeutic target. Importantly, JMJD5’s regulatory mechanisms show conservation across different physiological and pathological states, often involving epigenetic regulation of key gene expression or modulation of core protein stability via the demethylation-initiated enzymatic cascade to precisely control critical signaling pathways. This core regulatory paradigm provides a unified rationale for developing targeting strategies.

Despite significant progress in JMJD5 research in recent years, several key issues remain unresolved, which represent important directions for future investigation.

### 6.1 Mechanistic basis of JMJD5 functional heterogeneity

Elucidating the tissue-specific molecular mechanisms underlying the dual pro-oncogenic and anti-oncogenic properties of JMJD5 in various tumors remains a critical scientific question in this field. Based on existing evidence, we propose that the essence of JMJD5’s dual functionality does not stem from alterations in its intrinsic protein structure, but rather depends heavily on its distinct expression patterns across tissues, differentiated interaction protein networks, and specific tumor microenvironment (TME). This can be summarized into the following two dimensions:

1. Differences in specific expression patterns across cancer types: JMJD5 is often significantly overexpressed and exhibits oncogenic effects in breast cancer, prostate cancer, and oral squamous cell carcinoma (Wang et al., 2019; Song et al., 2022; Wang M. et al., 2025; Hsieh et al., 2023); conversely, its downregulation in lung cancer, hepatocellular carcinoma, and pancreatic cancer is closely associated with tumor suppressive phenotypes (Qu et al., 2022; Wang et al., 2022; Shen et al., 2023; Liu et al., 2023). Notably, relevant studies have confirmed that DNA hypermethylation-induced JMJD5 expression silencing has become a hallmark feature of hepatocellular carcinoma (Ahmed et al., 2024). Therefore, further investigation into the upstream mechanisms underlying the polarization of JMJD5 expression patterns across different cancer types is of paramount significance for elucidating the causes of its dual functions.
2. Tissue-dependent interactions between protein networks and downstream signaling: As a multifunctional regulatory factor, the final phenotype of JMJD5 is highly dependent on its tissue-specific interacting proteins. For instance, JMJD5 induces metabolic reprogramming through interaction with PKM2, exhibiting pro-oncogenic activity in breast cancer, prostate cancer, and glioblastoma (Wang et al., 2019; Song et al., 2022; Wang et al., 2014). Simultaneously, it promotes malignant progression in breast cancer and oral squamous cell carcinoma by enhancing CCNA1 transcription to drive cell proliferation (Zhao et al., 2015; Hsieh et al., 2023). A particularly notable example is its divergent regulation of the p53 signaling axis: in oral squamous cell carcinoma, JMJD5 inhibits apoptosis and induces epithelial-mesenchymal transition (EMT) via the p53/NF-κB axis to facilitate tumor invasion (Yao et al., 2019); whereas in lung cancer, it exerts tumor-suppressive effects by modulating the p53-TIGAR axis to alter metabolic pathways and suppress abnormal intracellular reactive oxygen species (ROS) accumulation (Liu et al., 2023). These differential downstream regulatory axes demonstrate that the functional reversal of JMJD5 depends on the primary signaling pathways and target molecules it interacts with under distinct tumor contexts.

In addition, other members of the JmjC family exhibit similar tissue-specific dual functions. Existing literature indicates that multiple family members, including JMJD1B, JMJD1C and JMJD3, possess both oncogenic and tumor suppressor properties (Lagunas-Rangel, 2021; Yoo et al., 2024). For instance, KDM3B (JMJD1B) was initially identified as a tumor suppressor gene against hematopoietic malignancies (Hu et al., 2001), but subsequent studies revealed its significantly upregulated expression as a critical risk factor for poor recurrence-free survival in non-small cell lung cancer (NSCLC) patients (Dalvi et al., 2017). Similarly, JMJD3 exerts both oncogenic effects by upregulating ALOX5 to promote gastric cancer progression and enhance ferroptosis sensitivity (Shu et al., 2025) and tumor suppressor effects by facilitating FOXO1 nuclear translocation to induce apoptosis in NSCLC (Ma et al., 2015). Researchers generally agree that the core mechanism underlying functional reversal of these JmjC family proteins is closely associated with tumor microenvironment heterogeneity and differential recruitment of downstream cofactors or signaling pathways.

### 6.2 Therapeutic targeting of JMJD5: opportunities and challenges

**FIGURE 9:**
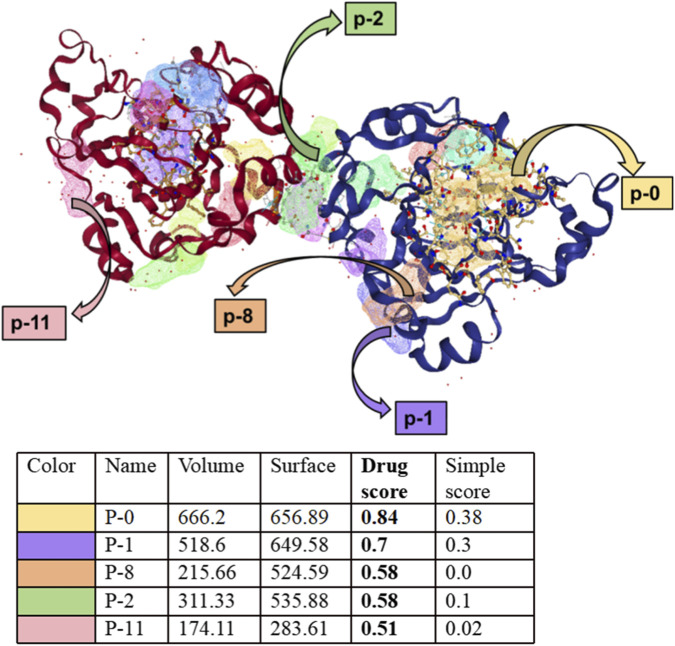
Analysis of potential drug-binding sites in the target protein. The figure shows the three-dimensional structure of the target protein and the quantitative analysis results of its potential drug-binding sites. In the three-dimensional structure diagram, different colors are used to mark five potential binding sites (P-0, P-1, P-8, P-2, P-11). The corresponding quantitative parameters of each binding site are shown in the table below: Volume: Represents the spatial volume of the binding site, with P-0 having the largest volume (666.2) and P-11 the smallest (174.11). Surface: Reflects the accessible surface area of the binding site, with P-0 showing the largest surface area (656.89). Drug score: Evaluates the potential of the binding site as a drug target. P-0 has the highest score (0.84), indicating it has the strongest potential for drug binding; followed by P-1 (0.7), P-8 and P-2 (0.58), and P-11 (0.51). Simple score: Assesses the basic binding capacity of the site, with P-0 (0.38) and P-1 (0.3) showing relatively higher values. Overall, the P-0 site (marked in yellow) has the largest volume, surface area, and the highest Drug score, making it the most promising drug-binding target in this protein.

The dual role of JMJD5 in cancer presents both unique opportunities and significant challenges for therapeutic development. From a druggability perspective, JMJD5 is considered a highly tractable target due to its well-defined, deep catalytic pocket that binds Fe (II) and α-KG, which is a classic druggable motif for small-molecule inhibitors (Staehle et al., 2021). Computational druggability analysis using the DoGSiteScorer tool gives JMJD5 a druggability score of 0.84 (out of 1.0), indicating excellent potential for small-molecule binding (Figure 9).

For cancers where JMJD5 acts as an oncogene (e.g., breast, prostate, oral squamous cell carcinoma), the development of selective small-molecule inhibitors is the most promising strategy. Currently, no JMJD5 specific inhibitors have been reported in the literature. The available pan-JmjC domain inhibitors, such as IOX1 (a broad spectrum α-KG competitive inhibitor) and GSK-J4 (a KDM6A/B inhibitor with weak activity against JMJD5), exhibit poor selectivity and significant off-target effects on other JmjC family members (e.g., KDM4, KDM5), leading to systemic toxicity (Chang et al., 2023; Dalpatraj et al., 2023; Monaghan et al., 2026). However, the high-resolution crystal structure of JMJD5 (PDB: 6F4N) reveals several unique structural features that can be exploited for selective inhibitor design: 1) A narrower substrate-binding pocket compared to other JmjC demethylases, which excludes larger substrates and can be targeted by shape-complementary molecules; 2) Unique α-KG binding residues (Tyr272 and Trp310) that form distinct hydrogen-bond networks with α-KG, which are not conserved in other JmjC proteins; 3) A hydrophobic subpocket adjacent to the Fe(II) binding site that is absent in most other JmjC family members. Structure-based rational drug design, combined with fragment-based high-throughput screening and covalent inhibitor development targeting the active site cysteine residues, is expected to yield the first generation of JMJD5-specific inhibitors in the next 3–5 years. In addition, proteolysis-targeting chimeras (PROTACs) that degrade JMJD5 protein are emerging as a promising alternative strategy, as they can achieve higher selectivity and potency than traditional enzyme inhibitors (Wang et al., 2024; Zhang et al., 2026).

For cancers where JMJD5 acts as a tumor suppressor (e.g., lung, liver, pancreatic cancer), therapeutic strategies should aim to restore JMJD5 expression or activity. Potential approaches include: (1) Epigenetic therapies to reverse JMJD5 promoter hypermethylation, such as low-dose DNA methyltransferase inhibitors (e.g., 5-azacytidine) and histone deacetylase inhibitors (Zhang et al., 2022; Dai et al., 2024); (2) Viral vector-mediated gene therapy to deliver JMJD5 expression vectors specifically to tumor cells; (3) Exosome-mediated delivery of recombinant JMJD5 protein, which has shown promising preclinical efficacy in lung cancer xenograft models with minimal systemic toxicity (Shen et al., 2023). However, a major challenge is the potential risk of off-target effects in normal tissues, as JMJD5 plays essential roles in embryonic development and tissue homeostasis. Therefore, tumor-specific delivery systems are critical for the clinical translation of these strategies.

Notably, the dual nature of JMJD5 necessitates strict precision medicine approaches: therapeutic interventions must be tailored to the specific cancer type and molecular subtype. For example, JMJD5 inhibitors should never be used in patients with lung or liver cancer, where JMJD5 functions as a tumor suppressor. Conversely, JMJD5 activators or gene therapy may accelerate tumor progression in breast or prostate cancer patients. This highlights the critical need for companion diagnostic tests (e.g., immunohistochemistry assays for JMJD5 protein expression) to stratify patients based on JMJD5 expression levels and functional status before initiating treatment.

### 6.3 Re-evaluation of JMJD5 enzymatic activities

A long-standing controversy surrounds the primary enzymatic activity of JMJD5: is it a lysine demethylase, an arginine hydroxylase, or a methyl-dependent protein hydrolase? Early studies (2010–2012) identified JMJD5 as an H3K36me2 demethylase, which shaped subsequent interpretations of its roles in cell cycle regulation, development, and circadian rhythms (Hsia et al., 2010; Jones et al., 2010; Ishimura et al., 2012). However, structural analyses later revealed that the substrate-binding pocket of JMJD5 is too narrow to accommodate methylated lysine residues, arguing against canonical demethylase function (Del Rizzo et al., 2012; Wang et al., 2013). Instead, biochemical studies confirmed arginine C-3 hydroxylase activity toward NFATc1 and RCCD1 (Wilkins et al., 2018; Youn et al., 2012). More recently, the discovery of methyl-dependent protein hydrolase activity further complicated this picture (Liu et al., 2017; Shen et al., 2017; Liu et al., 2020), as it could also explain the transcriptional effects previously attributed to demethylation.

To resolve these discrepancies, we propose a unifying hypothesis: substrate demethylation serves as an indispensable prerequisite and initiating step for JMJD5-mediated arginine hydroxylation and methylation-dependent proteolysis. In other words, JMJD5 executes its hydroxylation and proteolytic functions only after completing target demethylation. This demethylation-driven hierarchical enzymatic reaction underlies JMJD5’s functional diversity. We further propose that JMJD5 exhibits context-dependent activity: its substrate preference is determined by the cellular environment, co-factor availability, and interacting proteins. For example, under normoxic conditions with high α-KG levels, JMJD5 may favor arginine hydroxylation; under hypoxia with low α-KG levels, it may switch to protein hydrolase activity. Early observations of H3K36me2 demethylation likely reflect indirect effects (e.g., regulation of other demethylases) or non-specific activity in overexpression systems.

Mechanistically, we hypothesize that the three activities—demethylation, hydroxylation, and proteolysis—are linked via a common oxidative cascade. Initial demethylation generates a reactive intermediate that can subsequently undergo either hydroxylation or peptide bond cleavage depending on context. This view shifts from considering them as separate functions to understanding them as interconnected facets of a single oxidative regulatory mechanism. However, testing this hypothesis will require biochemical reconstitution with purified JMJD5 and defined methylated peptide substrates, as well as structural trapping of reaction intermediates.

Regarding the reported arginine demethylase activity of JMJD5, no convincing biochemical or structural evidence currently exists. Such claims rely largely on non-specific assays or overexpression systems, and no specific substrate or modification site has been unequivocally identified. Therefore, the existence of significant arginine demethylase activity under physiological conditions remains to be validated by future studies using rigorous biochemical and cellular approaches.

This integrated re-evaluation has important implications for future research: studies should employ catalytically inactive mutants that specifically abrogate individual enzymatic activities, rather than a single H321A/D323A mutant that abolishes all JmjC-dependent functions.

### 6.4 Other critical research gaps

In addition to the above issues, several other key questions remain to be addressed:

1. Limitations of current tumor models: Most existing studies rely on cell lines or simple xenograft models, which fail to recapitulate the complex *in vivo* tumor microenvironment (Crouigneau et al., 2024; de Visser and Joyce, 2023). Future studies should use patient-derived organoids and genetically engineered mouse models that more accurately reflect human cancer biology.
2. Clinical validation: The prognostic value of JMJD5 needs to be validated in large, multicenter clinical cohorts with long-term follow-up data. Additionally, the correlation between JMJD5 expression and response to specific therapies should be investigated.
3. JmjC family interactions: The functional relationships between JMJD5 and its close homologs JMJD6 and JMJD7 remain poorly understood. These proteins share similar enzymatic activities and may have redundant or compensatory functions in cancer (Liu et al., 2022; Liu et al., 2017; Liu et al., 2018).
4. Single-cell and spatial transcriptomics: Bulk transcriptomic analyses mask cellular heterogeneity. Future studies should use single-cell and spatial transcriptomics to map JMJD5 expression at the cellular level and investigate its role in tumor heterogeneity and the tumor microenvironment.

Based on the current research landscape, future investigations on JMJD5 should focus on resolving these fundamental questions, developing selective therapeutic agents, and validating its clinical utility as a prognostic biomarker and therapeutic target. Moreover, the proposed mechanistic link among the three enzymatic activities—if confirmed—would not only deepen our understanding of JMJD5 biology but also open new avenues for designing specific inhibitors that target the shared oxidative step or the unique downstream pathways.
